# The carbon footprint of modern imaging

**DOI:** 10.1097/MOU.0000000000001337

**Published:** 2025-09-03

**Authors:** Jan Vosshenrich, Elmar M. Merkle, Tobias Heye

**Affiliations:** Department of Radiology, University Hospital Basel, Basel, Switzerland

**Keywords:** carbon footprint, energy consumption, medical imaging, sustainability, urology

## Abstract

**Purpose of review:**

This review aims to highlight the often-overlooked environmental impact of medical imaging in urological practice, focusing on energy consumption, associated carbon emissions, and practical strategies for reducing the carbon footprint of imaging modalities.

**Recent findings:**

Medical imaging accounts for a significant proportion of a hospital's total energy use, with MRI, CT, and PET-CT being the most energy-intensive modalities. A recent life cycle assessment found that energy usage accounted for over half of a radiology department's greenhouse gas (GHG) emissions. Imaging systems such as fluoroscopy and ultrasound also contribute meaningfully, particularly when idle power consumption is overlooked. New data reveal that simple interventions, such as shutting down imaging devices during nonoperational hours and reducing unnecessary imaging, can cut nonoperational energy use by 20–70%.

**Summary:**

Given the slow adoption of energy-efficient imaging systems due to long development cycles, immediate emission reductions must come from operational changes. Strategies such as optimizing scheduling, shortening protocols, reduction of low-value imaging and powering down unused equipment can significantly reduce carbon emissions and costs – without compromising diagnostic value. Collaboration between referring clinicians and radiologists is critical to driving this transition.

## INTRODUCTION

Environmental sustainability has become an increasingly important consideration in healthcare, driven by rising energy costs, growing awareness of climate change, and the need to reduce greenhouse gas emissions. Medical imaging is a major contributor to the healthcare sector's carbon footprint – largely due to the high energy consumption of imaging equipment. Despite this, energy use has historically been overlooked in operational and clinical decision-making, as electricity costs were relatively minor, and energy consumption was rarely measured or reported. However, recent studies have highlighted the significant environmental impact of medical imaging, emphasizing the urgent need for more sustainable practices. In urology, where imaging is often integral to diagnosis, treatment planning, and follow-up, reducing the carbon footprint offers a tangible opportunity to contribute to climate goals without compromising patient care. This review explores current evidence, emissions data, and practical strategies for energy and emission reduction in urological imaging. 

**Box 1:**
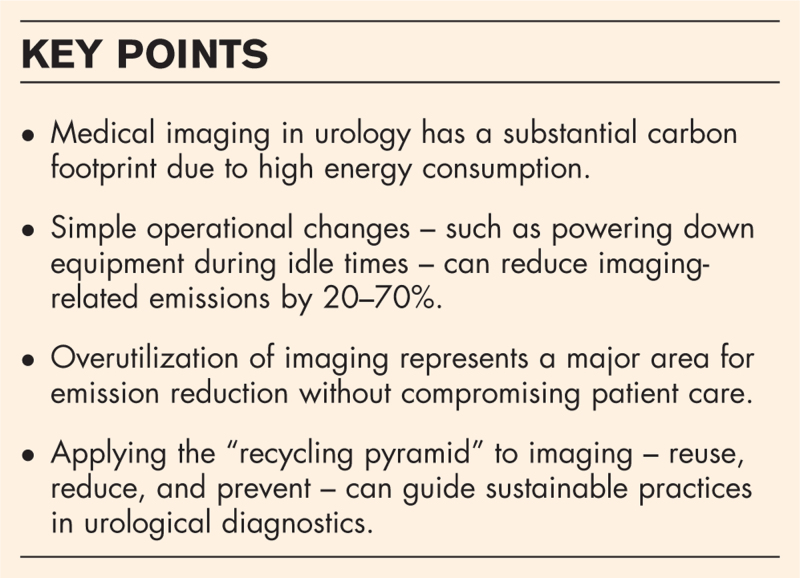
no caption available

## ENERGY – ELECTRICITY – CARBON INTENSITY

Energy consumption, in the form of electricity, was previously not a consideration in medical imaging since energy costs were relatively low. Electricity is not a resource which is typically measured or tracked and thus not made aware during clinical operations. This changed in the last five years due to the increasing awareness regarding sustainability in the healthcare sector [1] and an emerging energy crisis with rising energy prices partially driven by geopolitical tensions among other factors [2,3]. Considering the total energy consumption of a hospital, radiology is usually the specialty with the highest energy demand among all disciplines. Only the building infrastructure as a unit, including heating, ventilation and air conditioning (HVAC), is typically more energy intense [4]. Some estimations suggest that radiology may be responsible for up to 15% of the total electricity consumption of a hospital [5]. In one study, the operation of three CTs and four MRIs including adjunct cooling systems represented 4% of the total hospital energy consumption [6].

In a recent life cycle analysis of a radiology department by Thiel *et al.* the cumulative greenhouse gas (GHG) emissions over 10 years’ operating this department were calculated to add up to 4.6 kt of CO_2_ equivalent emissions, equivalent to almost 1100 gas-powered cars, annually [7^▪▪^]. The energy use operating modern medical imaging equipment accounted for 54% of total GHG emissions. Overall, MRI with 48% was the largest source of GHG emissions (17.5 kg CO_2_e/scan), followed by CT with 24% (9.2 kg CO_2_e/scan), X-ray (0.5–0.8 CO_2_e kg/scan)/fluoroscopy with 12%, and PACS/data storage with 12% [8]. To estimate the carbon emissions from electricity energy consumption, one can use the carbon intensity value of a respective region or country [9]. This value provides the amount of CO_2_ equivalent emissions per kWh of electricity. Overall, the average carbon intensity of electricity generation in the world is 0.473 kg CO_2_e/kWh. For Europe, this value is trending downwards, currently at 0.237 kg CO_2_e/kWh. The proportion of global clean electricity production is increasing currently at 41%, however at the same time the overall demand is rising and so are the associated GHG emissions [10]. Aside from the direct energy consumption for operating medical imaging equipment, water usage, adjunct cooling, waste and travel of staff and patients are considerable factors contributing to the overall carbon footprint. For example, adjunct cooling for MRI operation requires an additional 45% of the MRI energy consumption, a factor usually not included in GHG emission calculations [6].

## ENERGY CONSUMPTION AND CARBON EMISSIONS OF MEDICAL IMAGING

### Ultrasound

Ultrasound often represents the first-line imaging modality in urological practice, routinely employed in nearly all patient evaluations. A modern ultrasound system typically consumes around 2500 kWh of electricity per year – equivalent to approximately 50% of the annual electricity use and associated CO_2_ emissions of an average four-person household in Europe (in a detached home with electric water heating) [11]. This relatively modest energy consumption and carbon footprint (0.5 kgCO_2_e/scan) is largely attributable to the inclusion of efficient standby modes in contemporary devices [8]. In contrast, older ultrasound units lacking such energy-saving features demonstrate significantly higher carbon emissions.

### Fluoroscopy/radiography

Fluoroscopy is the other principal imaging modality frequently employed in urological diagnostics and therapeutic interventions. Due to the continuous heating required to maintain X-ray detector functionality, these systems exhibit notable energy consumption – even when powered off – ranging between 0.3 and 0.7 kW [12^▪^]. In idle mode, energy use increases to approximately 0.7–0.9 kW, though the energy demand remains substantially lower compared to advanced mono- and biplanar imaging radiology systems used in interventional radiology and cardiology that range at 4.5–7.6 kW [12^▪^]. Interestingly, the actual acquisition of fluoroscopic images contributes only minimally to total energy usage, as the power spikes are brief and infrequent [13]. To mitigate unnecessary energy expenditure, it is advisable to completely power down fluoroscopy units during off-hours. Additionally, the short boot-up and shutdown times of 2–5 min make it feasible to turn off systems during brief pauses, such as meal breaks or between procedures [12^▪^]. Overall, the annual carbon footprint of a fluoroscopy unit used in urology practice is comparable to that of approximately 1–1.5 four-person households.

### Computed tomography

Computed tomography (CT) scanners are usually located outside urology departments, but examinations are frequently requested for the diagnostic work-up and follow up of a variety of benign and malignant urological conditions. Furthermore, CT is integral for image-guided interventions such as biopsy, drainage, and nephrostomy placement. While concerns regarding radiation exposure have been extensively addressed over the past two decades, the environmental impact – particularly the carbon footprint – of CT imaging has only recently gained attention. Modern CT scanners consume approximately 20 000–35 000 kWh per year, corresponding to the annual energy usage of 4–7 four-person households [6,14]. To achieve meaningful reductions in both operational costs and environmental impact, radiology departments are advised to power down CT systems during periods of nonuse longer than one hour, as well as during off-hours, similar to interventional imaging systems [12^▪^,^14^]. However, this practice is currently not yet feasible with photon-counting CT scanners, as their advanced detector systems require over 12 h for proper calibration following shutdown.

### Magnetic resonance imaging

Magnetic resonance imaging (MRI) offers significant advantages in urology, particularly due to its superior soft tissue contrast and the absence of ionizing radiation. However, this diagnostic strength comes at the cost of exceptionally high energy consumption. State-of-the-art MRI scanners require approximately 80 000–170 000 kWh annually – equivalent to the annual energy usage of 16–34 four-person households [6,15]. Energy demands increase further with higher magnetic field strengths and larger bore sizes, thereby amplifying the associated carbon footprint. Notably, even when not in active use, MRI systems consume between 7 and 9 kW continuously, largely due to the need for sustained helium-based cooling [6,15]. Despite this baseline consumption, turning off MRI units during periods of inactivity is strongly recommended to modestly mitigate their substantial environmental impact. Additionally, the recent introduction of power save modes as well as advancements in image acceleration techniques and deep learning-based image reconstruction methods help to reduce the substantial carbon footprint of MRI [15,16^▪^].

### Nuclear medicine imaging

Scintigraphy and positron emission tomography-computed tomography (PET-CT) serve as key nuclear medicine imaging modalities for functional and molecular assessment of urological conditions. These techniques, while diagnostically invaluable – particularly in the context of renal dysfunction, urinary tract abnormalities, and oncologic staging – are associated with notable energy demands. Scintigraphy employs gamma cameras to detect gamma radiation from radiotracers, with the primary energy consumption stemming from continuous operation of detectors and image acquisition systems. PET-CT, which integrates the molecular imaging capabilities of PET with the anatomical resolution of CT, exhibits higher overall energy requirements. This is due to the use of high-energy positron-emitting radiopharmaceuticals – often cyclotron-produced – and the simultaneous operation of power-intensive CT scanners. There is currently limited data available regarding the energy consumption of nuclear medicine imaging. Published investigations only include estimations based on manufacturer-provided documentation for PET-CT and gamma cameras [17]. Based on measurements at the authors’ institution, the idle energy demand of PET-CT units ranges at around 6 kWh, equivalent to that of 1.5–2 CT scanners. Given complex calibration requirements for PET imaging, only the CT component of PET-CT systems can usually be shut down during off-hours, reducing the energy demand to around 4 kWh for the systems at the authors’ institution. Based on these measurements, PET-CT is estimated to have an annual energy consumption comparable to the energy usage of 10 four-person households.

### Picture archiving and communication systems and PC workstations

There is sparse data on picture archiving and communication systems’ (PACS) energy consumption and overall carbon footprint. However, a PACS system basically represents a data warehouse, that is typically high in energy and water consumption for cooling [4,7^▪▪^]. Shutting down personal computer (PC) workstations while not in use during off-hours allows to reduce the energy consumption and GHG emissions considerably, as demonstrated by a study using automatic shutdown protocols on 88 reporting workstations, resulting in annual savings of 3.4 metric tons CO_2_e and 17 MWh energy [18].

### Patient and staff travel

A frequently neglected aspect of the carbon footprint of medical imaging is the travel of patients and staff to and from the institution [19]. While there is of course the need for patients to travel to their appointments for imaging examinations, there is potential for savings by coupling imaging and clinical appointments on the same day. Similarly, airplane travel to international conferences by medical staff amount to considerable emissions, as demonstrated for an international radiology conference, totaling in 39 506 metric tons CO_2_e from 21 907 attendees, only 44.6% of the total number of attendees [20].

## OPTIMIZATION STRATEGIES AND SAVING POTENTIAL

Since energy consumption was not under consideration regarding optimization previously, there is an enormous potential for energy, cost, and carbon emission savings. Any nonproductive energy consumption from idle or high system off energy consumption of medical imaging equipment is energy waste and can be targeted for optimization. Turning off medical imaging equipment while not in use or during nonoperating hours at night or on weekends can result in reductions ranging from 20% to 70%. Most devices start up quickly and are operational within a few minutes if the need for emergency scanning arises. Implementing monitoring systems to understand the turning on/off routine of medical imaging devices within a department allows to identify devices which can be turned off during off-hours [21].

Identifying idle time during daytime operation and optimizing the examination schedule can lead to a reduction of up to 30% in consumption by shutting down devices earlier, as demonstrated for the operation of three CTs over one year [22]. While there are many opportunities to optimize the operation of a given imaging device [23,24] including shortening examination protocols to reduce the per patient carbon footprint and monitoring usage and consumption [21], addressing overutilization of imaging is another aspect with considerable saving potential.

It is estimated that 20–50% of medical imaging is of low value for the patient and thus unnecessary [25,26]. The diagnostic yield is a value illustrating how often a pathology is found when an examination is performed to check for this pathology. Imaging utilization has been increasing in the last decade [27], while diagnostic yield is declining as demonstrated for pulmonary embolism imaging [28].

Considering the recycling pyramid, which shows different measures and their impact in reducing resource waste, one can apply the pyramid to medical imaging, from lowest impact to highest:

Recover/Recycle: use prior imaging before initiating new imaging whenever feasible.

Reuse/Reduce: optimize energy consumption of medical imaging equipment by shutting down when not in use, shortening imaging protocols, enabling power save modes, switch from high carbon to low carbon imaging tests, for example, CT to ultrasound when feasible.

Prevent: reduce unnecessary imaging of low value for the patient.

As demonstrated, there are plenty of opportunities to reduce GHG emissions, save energy and costs in medical imaging without affecting the quality of care for patients. This win-win scenario is easy to achieve, representing the proverbial low hanging fruit while the saving potential is huge. Referring physicians and imaging specialists need to work together to initiate action on reducing the carbon footprint of modern medical imaging.

## CONCLUSION

Environmental sustainability has gained increased attention in recent years due to rising energy costs and the escalating impact of climate change. As medical imaging represents a substantial share of the healthcare sector's carbon footprint, there is growing pressure on manufacturers to develop more energy-efficient imaging systems. However, given typical development cycles of 5–8 years, widespread implementation of such innovations is unlikely before the end of the decade. In the interim, meaningful reductions in energy consumption – and thus carbon emissions – can be achieved at the institutional and individual level. Readily implementable strategies for reducing this impact include simple operational changes like powering down equipment during idle periods to systemic improvements in scheduling, protocol optimization, and reduction of unnecessary imaging. When applied thoughtfully, these measures may substantially reduce carbon emissions and operational costs without compromising diagnostic quality and patient care.

## Acknowledgements

*None.*

### Financial support and sponsorship

*No funding was received for this work.*

### Conflicts of interest

*There are no conflicts of interest.*

## REFERENCES AND RECOMMENDED READING

Papers of particular interest, published within the annual period of review, have been highlighted as:

▪ of special interest
▪▪ of outstanding interest
